# Comparison of Internal Fixations for Distal Clavicular Fractures Based on Loading Tests and Finite Element Analyses

**DOI:** 10.1155/2014/817321

**Published:** 2014-07-21

**Authors:** Rina Sakai, Terumasa Matsuura, Kensei Tanaka, Kentaro Uchida, Masaki Nakao, Kiyoshi Mabuchi

**Affiliations:** ^1^Department of Medical Engineering and Technology, School of Allied Health Sciences, Kitasato University, 1-15-1 Kitasato, Minami Ku, Sagamihara, Kanagawa 252-0373, Japan; ^2^Department of Orthopedic Surgery, Kitasato University, 1-15-1 Kitasato, Minami Ku, Sagamihara, Kanagawa 252-0373, Japan; ^3^Graduate School of Medical Science, Kitasato University, 1-15-1 Kitasato, Minami Ku, Sagamihara, Kanagawa 252-0373, Japan

## Abstract

It is difficult to apply strong and stable internal fixation to a fracture of the distal end of the clavicle because it is unstable, the distal clavicle fragment is small, and the fractured region is near the acromioclavicular joint. In this study, to identify a superior internal fixation method for unstable distal clavicular fracture, we compared three types of internal fixation (tension band wiring, scorpion, and LCP clavicle hook plate). Firstly, loading tests were performed, in which fixations were evaluated using bending stiffness and torsional stiffness as indices, followed by finite element analysis to evaluate fixability using the stress and strain as indices. The bending and torsional stiffness were significantly higher in the artificial clavicles fixed with the two types of plate than in that fixed by tension band wiring (*P* < 0.05). No marked stress concentration on the clavicle was noted in the scorpion because the arm plate did not interfere with the acromioclavicular joint, suggesting that favorable shoulder joint function can be achieved. The stability of fixation with the LCP clavicle hook plate and the scorpion was similar, and plate fixations were stronger than fixation by tension band wiring.

## 1. Introduction

Distal clavicular fracture is likely to undergo displacement because the proximal clavicle fragment tends to be pulled upwards by the trapezius and the distal fragment tends to be pulled downwards [1]. Furthermore, since the distal fragment is small in cases of distal clavicular fracture and the fracture occurs at a site closer to the acromioclavicular joint, firm fixation of the fracture is difficult. According to the Craig classification of distal clavicular fracture, types IIa and IIb are unstable and conservative treatment is likely to result in nonunion, thus often requiring surgery [2].

Although diverse operative procedures have been applied to surgical treatment of Craig type IIa and IIb cases, it is difficult to achieve stable and firm fixation while avoiding interference with the acromioclavicular joint [3–7]. Although tension band wiring has been conventionally applied to fix distal clavicular fractures [3], our university hospital has been using, since 2000, Scorpion (Aimdeic, Japan) as a means of fixing unstable distal clavicular fracture without crossing the acromioclavicular joint. The plate used for this technique is thin (2 mm) and has an anatomical design curved along the shape of the clavicle. Another technique of fixation uses an LCP clavicle hook plate (DePuy Synthes, IN, USA), which is a fracture plate designed to apply a characteristic hook across the acromioclavicular joint [8]. This plate is thick and therefore has high stiffness. For the screws, locking screws were used.

Loading test is useful for comparison among different methods of internal fixation of distal clavicular fracture [9]. Kanda et al. conducted a bending test based on the assumption that the proximal clavicle fragment is pulled upwards by the trapezius and the distal fragment is pulled downwards by the arm's weight [1]. When the shoulder is raised, not only scapular rotation but also clavicular rotation occurs [10], and evaluation of the validity of internal fixation of distal clavicular fracture requires not only a bending stiffness test but also a torsional stiffness test. To date, however, few reports of such evaluation have been published.

The LCP clavicle hook plate has been reported to induce bone resorption of the area where the hook on the subacromial plane touches the bone [11]. One possible cause of this phenomenon is formation of local/stationary stress between the hook and the bone, and a similar problem can occur between the arm and the bone when Scorpion is used. Finite element analysis is useful as a means of clarifying the stress in such cases. If the mechanostat theory is applied to the stress and strain thus determined, it will be possible to predict whether the fixed area is undergoing bone resorption or ossification [12].

The present study was designed to identify the best technique of fixation for distal clavicular fracture from among 3 methods (tension band wiring, Scorpion, and LCP clavicle hook plate) through evaluation of bending stiffness and torsional stiffness. The study additionally involved analysis of the bone status and fixability, with stress and strain serving as indicators, by carrying out finite element analysis on the two kinds of plate shown to have no difference in the loading test data.

## 2. Materials and Methods

### 2.1. Loading Test

**Figure 1:**
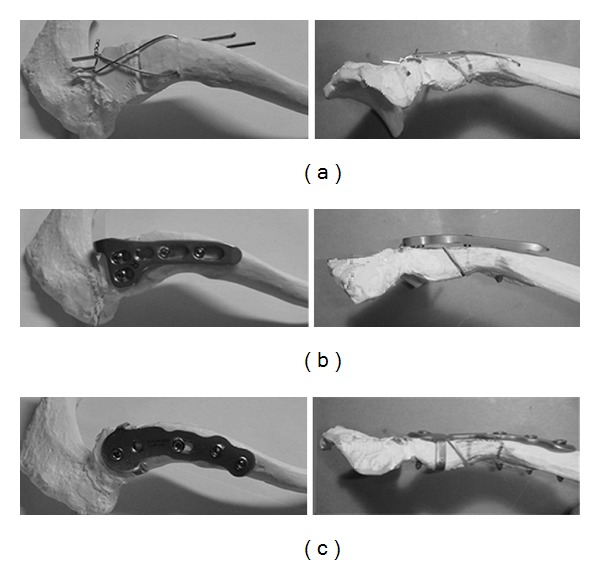
Internal fixations. (a) Tension band wiring. (b) LCP clavicular hook plate. Longer wires were used in order to clearly present the insertion angles and positions. (c) Scorpion.

Tension band wiring using 1.8 mm K-wire and 0.91 mm low-carbon steel wire (Mizuho, Japan), Scorpion (Ai-medic, Japan), and the LCP clavicle hook plate (DePuy Synthes, IN, USA) using locking screws were investigated. For the artificial clavicle, SAWBONES Left Shoulder 1020-20 (Pacific Research Laboratory, WA, USA) with physical properties similar to those of biological bone was used, and the 3 internal fixations were applied employing the standardized procedures. After internal fixation, a fracture line was prepared from a site 20 mm proximal from the distal end over a site 30 mm proximal from the proximal end of the clavicle (Figure 1). Fracture of Craig type IIa was artificially created for this study [2]. Artificial clavicles without a fracture were regarded as a control.

**Figure 2:**
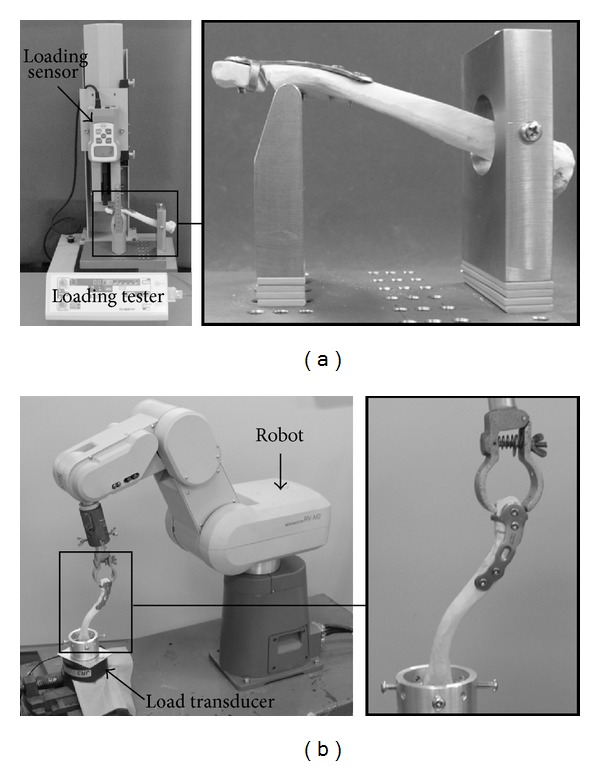
Measurement device overview. (a) Loading tester and loading sensor. Position of test bench and specimen. (b) An arm type robot and load transducer. Position of specimen.

The prepared clavicular fracture model was placed on the test bench, and a bending test was performed by applying a loading force to the distal end of the clavicle. The loaded force reproduced downward traction of the distal clavicle fragment induced by the upper limb weight, and the support of the test bench reproduced upward traction of the proximal clavicle fragment caused by the trapezius muscle [4]. The test bench was installed in a loading tester, FGS-50VB-L, H (SHIMPO, Japan), and a force was loaded on the distal end of the clavicle in the downward direction using a digital load sensor, FGP-100 (SHIMPO, Japan) (Figure 2(a)). Since the upper limb weight accounts for 5% of the body weight, that is, the clavicle is weighted downward by a 30–40 N force of gravity, the force was set to a maximum of 50 N, higher than the traction by the upper limb. In a torsion test, rotation of the clavicle with rotation of the scapula in shoulder elevation was simulated [3]. Using a five-axis multijoint arm-type robot RV-M2 (Mitsubishi Electric, Japan) connected to a general-purpose computer, the test was programmed using QUICK BASIC. Numerical sequence commands were sent to the robot so that the robot arm held the distal end of the clavicle and rotated 10°. The angle of 10° refers to the angle of clavicular rotation when the arm is swung when walking [10]. The proximal end of the clavicle was fixed to a 6-axis strain gauge-type load transducer, CA95776 (JR3 Inc., NC, USA), and the torque was measured (Figure 2(b)).

The bending and torsional stiffness levels of the fracture region were compared among clavicles fixed employing the three fixation methods. To measure the displacement for calculating the stiffness, photographs including a standard measure were taken before and after loading, and displacement of the distal end was measured using image analysis software, Image J (NIH, MD, USA). In statistical analysis, one-way ANOVA and between-group multiple comparisons employing the Tukey-Kramer method were performed.

### 2.2. Finite Element Analysis

**Figure 3:**
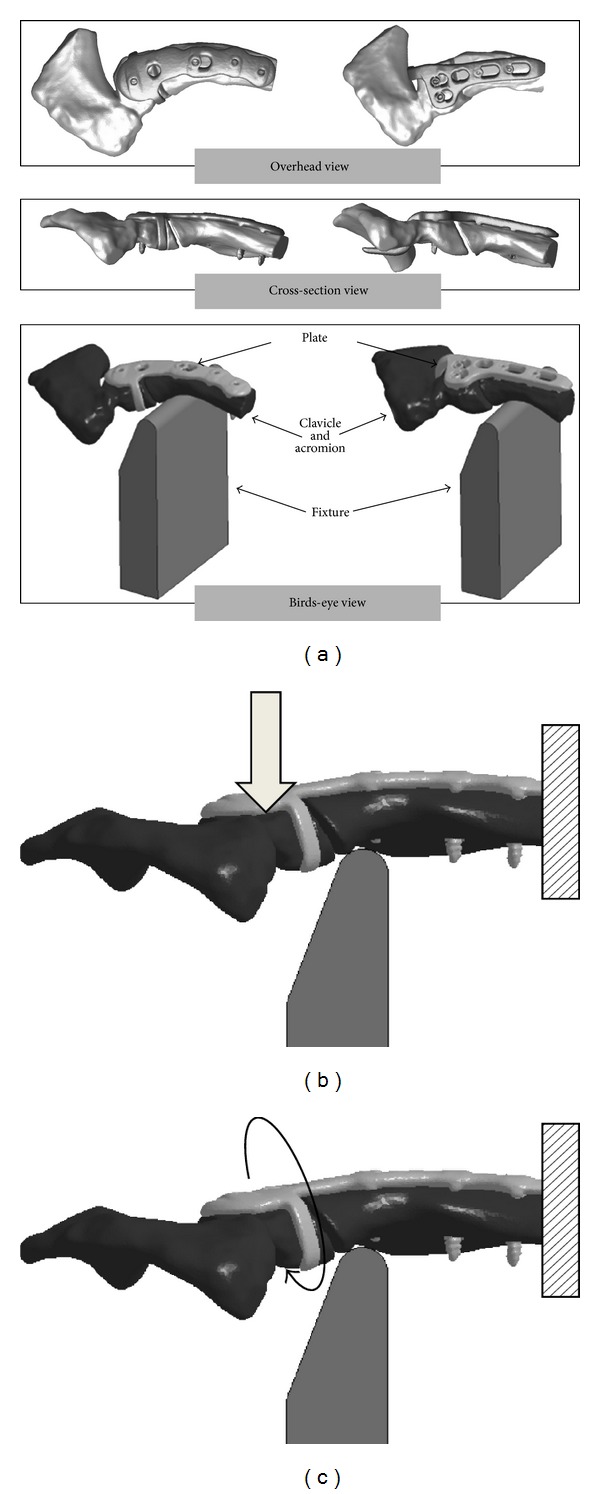
Finite element models, loading and constraint conditions. (a) Left is LCP clavicular hook plate model. Right is Scorpion model. (b) Applied vertical load was 50 N on the distal end of the clavicle. Squares show constraint. (c) Distal end of the clavicle was rotated 10°. Squares show constraint.

For the creation of finite element models, artificial clavicles with a Scorpion or an LCP clavicle hook plate attached were photographed with a *μ*CT device (inspeXio SMX-90CT, Shimazu, Japan). These images were processed with an image processing program, Simpleware (JSOL, Japan), to create mesh models. When models were created, the 3 parts (acromion, clavicle, and plate) were prepared with trihedral elements. Each of the hook plate model and the Scorpion model consisted of about 200,000 elements (Figure 3(a)). As constants for the materials, elastic modulus, Poisson's ratio, and mass density [13, 14] were defined (Table 1).

The hardware used for analysis was Endeavor Pro-4500 (EPSON, Japan). The finite element analysis code LS-DYNA ver. 971 (Terrabyte, Japan) was employed as the solver. Loading was carried out under two settings (vertical loading and rotation) similar to the measurement of stiffness. Vertical loading was carried out by applying a 50 N load to the distal end of the clavicle to induce downward traction of the distal bone fragment with the arm's weight (Figure 3(b)). Rotation involved 10° rotation of the clavicle around the acromion, modeling after clavicular rotation seen at the time of shoulder raising (Figure 3(c)). Immobilization involved complete immobilization of the clavicular diaphysis. Each of stress and strain was measured as the average of displacements at 10 nodal points selected at random.

## 3. Results

**Figure 4:**
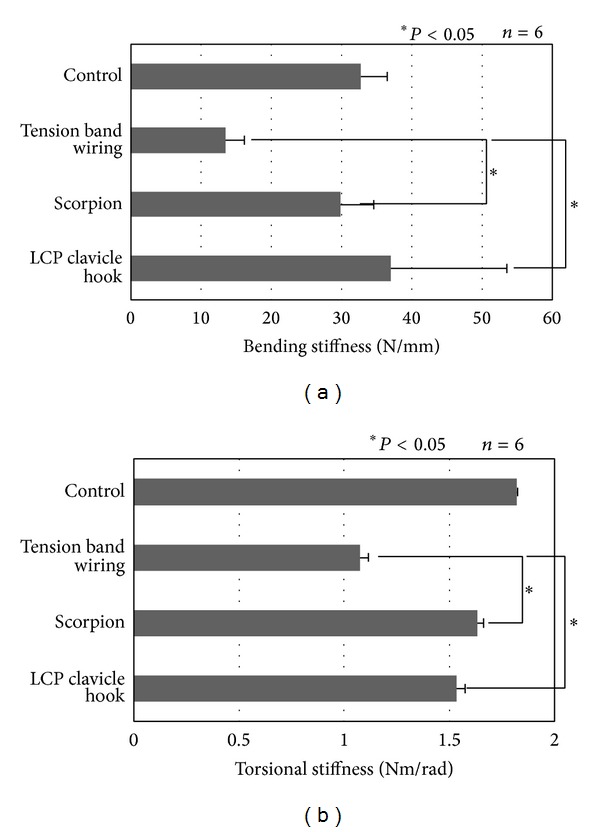
Bending and torsional stiffness of internal fixations. (a) Bending stiffness. (b) Torsional stiffness.

**Figure 5:**
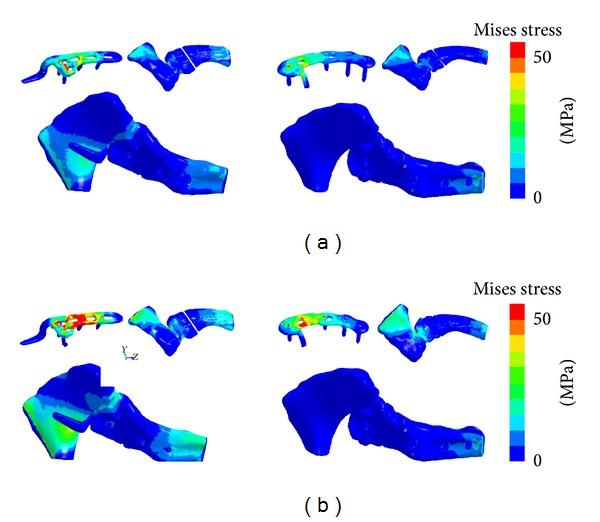
von Mises stress distribution. Left are LCP clavicular hook plates. Right are Scorpions. (a) Vertical load. (b) Rotational load.

The levels of bending stiffness of artificial clavicles fixed with tension band wiring, a Scorpion, and an LCP clavicle hook plate were 13.4 ± 2.8, 30.0 ± 4.7, and 37.0 ± 17.0 N/mm, respectively (Figure 4(a)). The levels of torsional stiffness of artificial clavicles fixed with tension band wiring, a Scorpion, and an LCP clavicle hook plate were 1.07 ± 0.04, 1.63 ± 0.03, and 1.53 ± 0.04 N/mm, respectively (Figure 4(b)). The levels of bending and torsional stiffness were significantly higher in the artificial clavicles fixed with the two types of plate than in that fixed by tension band wiring (*P* < 0.05). No significant difference was noted in the levels of bending and torsional stiffness between the clavicles fixed with a Scorpion and an LCP clavicle hook plate.

von Mises stress during vertical loading was 1 MPa at the distant clavicle in contact with the Scorpion arm and 13.3 MPa on the subacromial plane in contact with the hook of the LCP clavicle hook plate (Figure 5(a)). Maximum stress at the clavicular diaphysis was higher with the LCP clavicle hook plate (13.3 MPa) than with the Scorpion (8.5 MPa). During rotation, von Mises stress was 8.7 MPa at the distal end of the clavicle in contact with Scorpion and 10.2 MPa at the bone between the subacromial plane and the hook of the LCP clavicle hook plate (Figure 5(b)).

During vertical loading, von Mises strain was 700 *μ* at the distal end of the clavicle and the acromion in contact with the Scorpion and 800 *μ* at the same point in contact with the LCP clavicle hook plate. During rotation, von Mises strain was 790 *μ* at the distal end of the clavicle and the acromion in contact with the Scorpion and 921 *μ* at the same point in contact with the LCP clavicle hook plate (Table 2). Thus, under each setting of loading, von Mises strain on the bone fixed with the LCP clavicle hook plate was higher than the equivalent strain on the bone fixed with the Scorpion. The equivalent strain on the plate was lower during torsional loading in the vicinity of the LCP clavicle hook plate than the strain during fixation with the Scorpion.

## 4. Discussion

The principle of tension band wiring is prevention of displacement of the fracture region by using wire and conversion of the extension force of the wire to compression force, and that of plate fixation is prevention of displacement of the fracture region by using a plate and compression force of screws [3]. Since a compression force was loaded vertically to the axis of the bone in the bending test, considering the stiffness of wire and plates, it is reasonable that the bending stiffness with tension band wiring was lower than those with the plate fixations [1]. In the torsion test, torque was generated at the distal end of the clavicle, through which the distal and proximal clavicle fragments were jointed by pressing them other, producing an extension force in the plate and wire. Wire more readily extends than plates, which may have been the reason for the lower torsional stiffness of tension band wiring than those of the plate fixations. The 2 wires of tension band wiring applied in the experiment were longer than those applied in clinical cases in order to clearly present the insertion angles and positions, and this condition is unlikely to influence the stiffness.

The absence of a marked difference in the bending stiffness between the plate types may have been due to the absence of a major difference in the force of screws or clavicle-holding force. Two locking screws each were used for the distal and proximal clavicle fragments for fixation with the LCP clavicle hook plate. Application of the locking screws may have generated marked compression, which firmly fixed the fracture region with the plate. In fixation with the Scorpion, the distal clavicle fragment was jointed by pressing by the arm and fixed with a screw, and the proximal clavicle fragment was fixed with three screws. The distal clavicle fragment was held at the anteroposterior surfaces by the arm, which may have generated marked compression and firmly fixed the fracture region. It was clarified that there was no marked difference between the fixability of locking screws of the LCP clavicle hook plate and that of jointing by pressing by the arm of the nonlocking plate, yielding an equivalent effect.

Although the LCP clavicle hook plate has been reported to induce bone resorption in the subacromial region [11], the stress at the hook of the LCP clavicle hook plate and on the subacromial plane is unlikely to pose a significant problem immediately. Regarding the balance between bone resorption and ossification, it has been reported that the minimum strain causing switching to ossification is 300 *μ* [15]. Because the strain was 300 *μ* or higher during use of each plate and under each setting of loading in the present study, we speculate that each plate tested can stimulate bone growth. However, in view of a report that bone resorption occurred in clinical cases during fixation with the LCP clavicle hook plate, it seems undesirable to keep this plate inserted for a long period of time.

It has been reported that wires of tension band wiring migrated and caused serious complications [16]. Range of motion training after treatment with tension band wiring is limited for 3–6 months after surgery because the wire may be dislocated by rotation of the clavicle, but training can be started the day after surgery when an LCP clavicle hook plate or Scorpion is used. Range of motion training can be started earlier by selecting the plate fixation method, rather than tension band wiring, which may facilitate the earlier recovery of shoulder movement. When tension band wiring is retained for a prolonged period, nail removal is essential because of concern over the breakage of deteriorated wire [3]. The plate is thick and the hook is sharp in an LCP clavicle hook plate, for which the removal of nails is necessary because of concern over displacement. In contrast, the Scorpion is thin and curved along the clavicular shape, for which the removal of nails is not necessarily essential, and the possibility of infection and physical burden may be subsequently reduced.

This study involved the following limitations. First, because the fracture model was based on a Craig type II fracture, the results and discussions in this study are valid only for such fractures. Second, clavicle growth over time was not taken into account when analyses were carried out. Since this was a simple load test in one direction, to discuss the modes of failure, it is necessary to improve the loading method and perform a test applying a complex load employing several modes. Therefore, the results from this study enable prediction of only initial fixation performance (including prediction of the status of ossification only for the early stage of fixation). Compressive loading from the acromion is a possible cause of fracture of the distal end of the clavicle, and future studies on this factor are also desirable [17].

## Figures and Tables

**Table 1:** Material properties of clavicle, acromion, plates, and fixture.

	Mass density	Elastic	Poisson
	(ton/mm^3^)	coefficient (MPa)	ratio (—)
Clavicle and acromion	2.0*e* − 9	10,000	0.3
Plates (titanium)	4.5*e* − 9	100,000	0.3
Fixture (rigid body)	4.5*e* − 9	—	—

**Table 2:** Mises strain (*μ* strain) on clavicle, acromion, and plates.

	Scorpion		LCP clavicle hook	
	Bending	Torsional	Bending	Torsional
Distal clavicle	700	790	800	921
Shaft of clavicle	350	790	400	921
Acromion	700	790	800	921
Distal plate	222	300	800	300
Proximal plate	100	256	800	150
